# The Mitochondrial Complex I Activity Is Reduced in Cells with Impaired Cystic Fibrosis Transmembrane Conductance Regulator (CFTR) Function

**DOI:** 10.1371/journal.pone.0048059

**Published:** 2012-11-21

**Authors:** Angel G. Valdivieso, Mariángeles Clauzure, María C. Marín, Guillermo L. Taminelli, María M. Massip Copiz, Francisco Sánchez, Gustavo Schulman, María L. Teiber, Tomás A. Santa-Coloma

**Affiliations:** Institute for Biomedical Research, Laboratory of Cellular and Molecular Biology, School of Medical Sciences, Pontifical Catholic University of Argentina (UCA) and The National Research Council of Argentina (CONICET), Buenos Aires, Argentina; University of Tübingen, Germany

## Abstract

Cystic fibrosis (CF) is a frequent and lethal autosomal recessive disease. It results from different possible mutations in the *CFTR* gene, which encodes the CFTR chloride channel. We have previously studied the differential expression of genes in CF and CF corrected cell lines, and found a reduced expression of *MTND4* in CF cells. *MTND4* is a mitochondrial gene encoding the MTND4 subunit of the mitochondrial Complex I (mCx-I). Since this subunit is essential for the assembly and activity of mCx-I, we have now studied whether the activity of this complex was also affected in CF cells. By using Blue Native-PAGE, the in-gel activity (IGA) of the mCx-I was found reduced in CFDE and IB3-1 cells (CF cell lines) compared with CFDE/6RepCFTR and S9 cells, respectively (CFDE and IB3-1 cells ectopically expressing wild-type CFTR). Moreover, colon carcinoma T84 and Caco-2 cells, which express wt-CFTR, either treated with CFTR inhibitors (glibenclamide, CFTR(inh)-172 or GlyH101) or transfected with a CFTR-specific shRNAi, showed a significant reduction on the IGA of mCx-I. The reduction of the mCx-I activity caused by CFTR inhibition under physiological or pathological conditions may have a profound impact on mitochondrial functions of CF and non-CF cells.

## Introduction

Cystic fibrosis (CF) is an autosomal recessive disease caused by mutations in the *CFTR* (Cystic Fibrosis Transmembrane Conductance Regulator) gene. This gene was cloned in 1989 [1], [2] and soon identified as a chloride channel [3], [4]. More than 1,900 possible mutations have been identified so far (www.genet.sickkids.on.ca)[5], which impair the expression of the *CFTR* mRNA, the traffic of its protein product towards the cell membrane or alter its turnover anddegradation [6], [7], [8], [9]. Before the *CFTR* gene was cloned, several reports suggested a possible mitochondrial failure associated to CF. Burton L. Shapiro and colleagues found that CF cells are more sensitive to the Complex I (NADH:ubiquinone oxidoreductase, mCx-I, mitochondrial Complex I, EC 1.6.5.3) inhibitor rotenone and consume more oxygen than normal cells [10]. They also found altered optimal pH and Km values for this mitochondrial enzyme [11], as well as an elevated calcium uptake, in CF mitochondria, the latter attributed to a possible defect in the respiratory chain [12]. Based on these results, these authors postulated that the gene affected in CF might be a component of the mitochondrial Complex I [10], [11]. However, after CFTR was identified as a membrane protein with chloride transport activity (chloride channel), the mitochondrial hypothesis was disregarded and no further work was done for many years on the subject. Possible indirect effects of CFTR or Cl^−^ over mitochondria were not considered as a possibility at that time and until recently, no further studies suggested that the CFTR failure could indirectly lead to a mitochondrial failure [13], [14], [15], [16].

By using differential display, we have previously studied the differential expression of genes in CF and non-CF cells, and identified several “CFTR-dependent genes”, including *c-Src* [17], *MUC1* [17], *CISD1* [14] and *MTND4* [15]. We first studied one spot that was increased in CF cells and resulted to be c-Src. Then, we selected two spots that, contrary to c-Src, were clearly reduced in CF cells. Noteworthy, both genes, *CISD1* [14] and *MTND4* [15], codified for mitochondrial proteins. CISD1 was also found by Colca et al. [18] as a mitochondrial receptor for pioglitazone, and was named by them mitoNEET. The exact function of CISD1 is unknown yet. It has been recently proposed that CISD1 might act as a redox sensor, as a modulator of oxidative phosphorylation (OXPHOS), or as a carrier of [2Fe2S] clusters to apoproteins acceptors into mitochondria [19], [20], [21], [22], [23], [24], [25], [26]. On the other hand, *MTND4* encodes the MTND4 subunit of mitochondrial Complex I (mCx-I). This complex is the entry point of electrons to the OXPHOS system, transferring electrons from NADH (reduced nicotinamide adenine dinucleotide) to ubiquinone; the electron transference is coupled to the proton pumping inside the mitochondrial intermembrane space (IMS) to form, in part, the proton gradient used to produce ATP [27], [28]. Interestingly, MTND4 is essential for the assembly and proper activity of the mCx-I [29]. Different mutations within the *MTND4* gene result in a loss of enzyme activity [30], as evidenced in Leber's hereditary optic neuropathy (LHON) disease [31], [32], [33]. This condition is a maternally inherited form of central vision loss in which the mCx-I activity is impaired.

**Figure 1:**
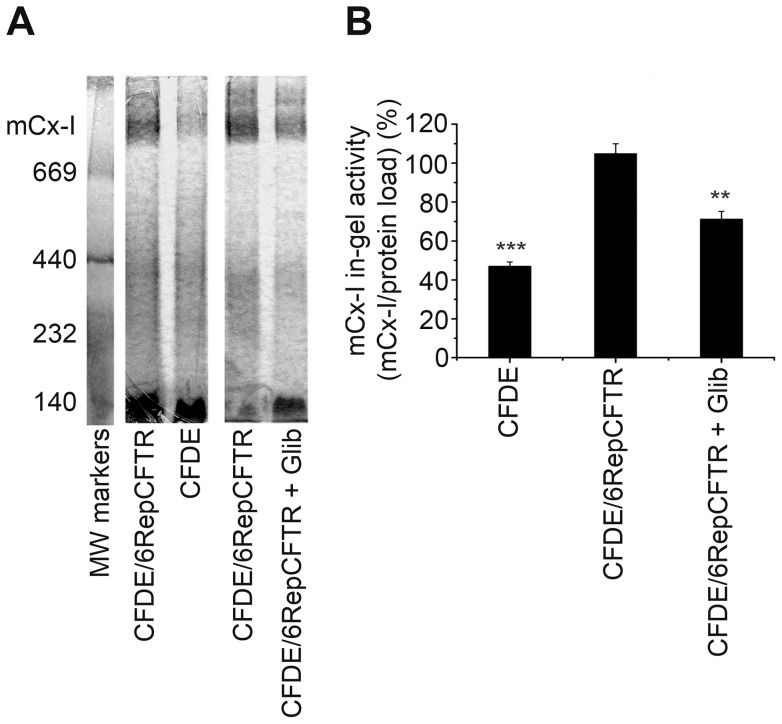
Mitochondrial complex I in-gel activity (IGA) of CFDE and CFDE/6RepCFTR cells. A: IGA of mitochondrial extracts from CFDE (CF cells), CFDE/6RepCFTR cells (rescued cells ectopically expressing wt-CFTR), and the same cells treated with glibenclamide, a CFTR chloride transport inhibitor. B: Densitometric quantification and statistical analysis of the results shown in panel A. IGA was calculated as the ratio (mCx-I activity)/(protein load), both expressed as arbitrary units. The average activity of the mCx-I in CFDE/6RepCFTR cells was considered 100%. Measurements were performed in duplicate and data are expressed as mean ± SE of three independent experiments (n = 3). ** indicates p<0.01 and *** indicates p<0.001, referred to CFDE/6RepCFTR cells.

The reduced expression of *MTND4* found in CF cells [15], and the important role of this subunit in mCx-I assembly, prompted us to test whether the activity of this complex was affected in CF cells or in cells with impaired CFTR activity (by using CFTR channel inhibitors or shRNA). We show here that the in-gel activity (IGA) of mCx-I was reduced in CF cells. Moreover, this activity can be modulated in cells expressing wt-CFTR in the presence of CFTR inhibitors or shRNA, demonstrating a causal effect between the CFTR activity and the mCx-I activity. The results are in agreement with earlier reports on mitochondrial alterations in CF observed more than two decades ago [10], [11], [12], [34], [35], [36], [37], which were later erroneously disregarded when the CFTR was found to be a chloride channel.

**Figure 2:**
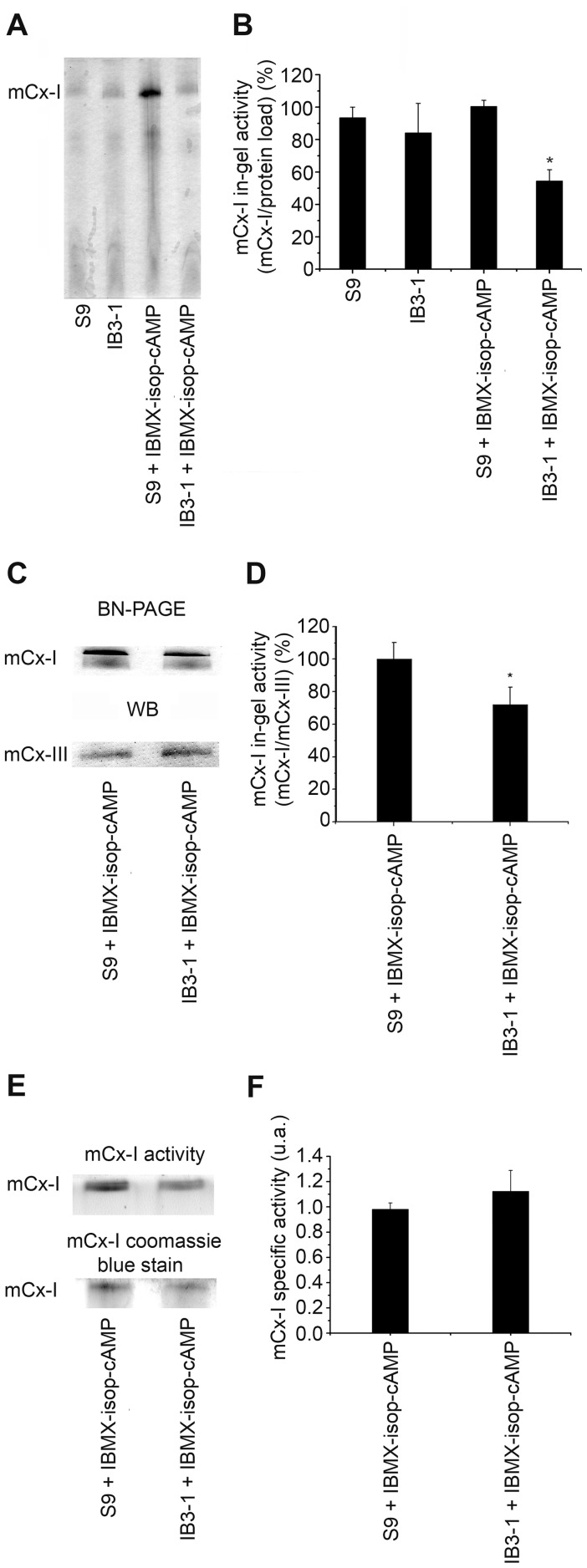
Mitochondrial complex I in-gel activity (IGA) of IB3-1 and S9 cells. A: IGA of mitochondrial extracts from Control and CFTR-stimulated IB3-1 and S9 cells (IBMX-isop-cAMP), adding 200 µM cAMP, 10 µM isoproterenol, 200 µM IBMX, for 24 h. B: Densitometric quantification and statistical analysis of the results shown in panel A. IGA was calculated as indicated in Figure 1. Measurements were performed in duplicate and data are expressed as mean ± SE of five independent experiments (n = 5). * indicates p<0.05, as compared with S9 stimulated cells. C: IGA of mCx-I and mCx-III (UQCRC1) expression measured by using Western blots from S9 and IB3-1 cells (both after CFTR stimulation). D: Densitometric quantification and statistical analysis of the results shown in panel C. IGA of mCx-I was calculated as the ratio mCx-I IGA/UQCRC1. Measurements were performed in duplicate and data are expressed as mean ± SE of two independent experiments (n = 2). * indicates p<0.05, as compared with S9 stimulated cells. E: IGA of the mCx-I and Coomassie blue stain from a BN-PAGE using mitochondrial extracts from S9 and IB3-1 cells. F: Specific activity of the results shown in panel E, calculated as the ratio mCx-I IGA/mCx-I coomassie blue stain. The mCx-I specific activity is expressed in arbitrary units (a.u.) as mean ± SE (n = 3). The specific activity of CF cells (IB3-1) and CF corrected cells (S9) showed similar values, without significant differences (p>0.05).

## Materials and Methods

### Materials

Bovine fibronectin, collagen Type I, pepstatin, PMSF (phenylmethylsulfonyl fluoride), leupeptin, glibenclamide, dimethyl sulfoxide (DMSO, culture grade), NADH, dibutyryl-cAMP, lauryl maltoside, IBMX (3-isobutyl-1-methyl xanthine), (−)-isoproterenol hydrochloride and valinomycin were purchased from Sigma-Aldrich (St. Louis, MO). Cytochrome c, CFTR(inh)-172 (5-[(4-Carboxyphenyl) methylene]-2-thioxo-3-[(3-trifluoromethyl)phenyl-4-thiazolidinone) and GlyH101 (N-(2-naphthalenyl)-[(3,5-dibromo-2,4-ihydroxyphenyl)methylene]glycine hydrazide) were from Calbiochem (San Diego, CA). Aminocaproic acid (6-aminohexanoic acid) and nitroblue tetrazolium (NBT) were from Fluka (Sigma-Aldrich). NBT-BCIP (BCIP, 5-bromo-4-chloro-3′-indolyphosphate) were from Promega (Madison, WI), Coomassie brilliant blue G-250 and R-250 were supplied by Bio-Rad Laboratories (Hercules, CA). Trypsin was purchased from Life Technologies (GIBCO BRL, Rockville, MD) and SPQ (6-methoxy-N-[3-sulfopropyl]quinolinium) from Invitrogen (Carlsbad, CA). All other reagents were analytical grade.

**Figure 3:**
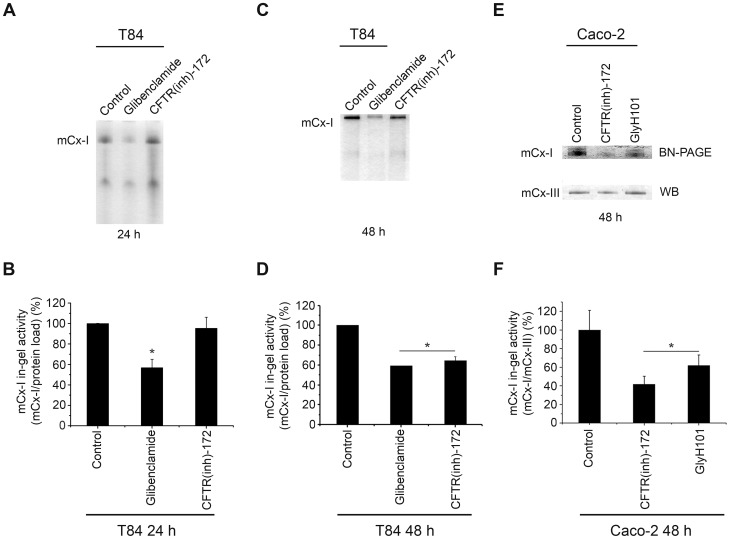
Mitochondrial complex I in-gel activity (IGA) measured in cells expressing wt-CFTR. A: IGA of mitochondrial extracts from T84 cells after 24 h of treatment with 100 µM glibenclamide or 5 µM CFTR(inh)-172. B: Densitometric quantification of the results shown in panel A, expressed as % ratio of (mCx-I activity)/(protein load). C: IGA of mitochondrial extracts from T84 cells after 48 h of treatment with 100 µM glibenclamide or 5 µM CFTR(inh)-172. D: Densitometric queantification of C. E: IGA of the mCx-I from Caco-2 cells after 48 h of treatment with 5 µM GlyH101 or 5 µM CFTR(inh)-172, and WB of the mCx-III subunit UQCRC1, as internal standard. F: Densitometric quantification of the results shown in E expressed as % ratio of (mCx-I IGA)/UQCRC1(a.u.). The activity of mCx-I in T84 and Caco-2 cells treated with the same amount of DMSO (0.1%) was considered as 100%. Measurements in T84 cells were performed in duplicate and data are expressed as mean ± SE of three independent experiments (n = 3). Caco-2 cells results were obtained in triplicate and expressed as mean ± SE of three independent experiments (n = 3). * indicates p<0.05, as compared with control cells (ANOVA and Turkey's test).

### Cell culture

CFDE, CFDE/6RepCFTR, IB3-1, S9, T84 and Caco-2 cells were used in the experiments. CFDE and CFDE/6RepCFTR cells were a gift from Dr. Dieter C. Gruenert (UCSF). IB3-1 (CRL-2777), S9 (CRL-2778), T84 (CCL-248) and Caco-2 (HTB-37) cells were purchased from ATCC (www.atcc.org). CFDE cells are tracheobronchial cells derived from a CF patient with an unknown genotype (the most frequent mutations are absent; unpublished observations); CFDE/6RepCFTR are CFDE cells that ectopically express the wt-CFTR [38]. IB3-1 cells are bronchial epithelial cells derived from a CF patient that exhibited the most frequent mutation, ΔF508. These last cells have been immortalized using the hybrid adenovirus adeno-12-SV40 [39]. S9 cells are IB3-1 cells transduced with an adeno-associated viral vector to stably express wt-CFTR [40]. Finally, T84 and Caco-2 cells are human colon carcinoma epithelial cells that express wt-CFTR [41], [42], [43]. All cell lines were cultured in DMEM/F12 (Life Technologies, GIBCO BRL, Rockville, MD) supplemented with 10% FBS (BIOSER, Buenos Aires, Argentina), 100 units/ml penicillin, 100 µg/ml streptomycin, and 0.25 µg/ml amphotericin B (Life Technologies, GIBCO BRL, Rockville, MD). Cultures were grown in a humidified atmosphere containing 5% CO_2_. Additionally, CFDE/6RepCFTR cells were grown in the presence of 50 µg/ml hygromycin B (Sigma-Aldrich), in order to select and maintain cells that ectopically expresses wt-CFTR. All cells were plated at a density of 15×10^3^ cells/cm^2^. Before the assays, the cells were cultured 24 h in serum-free medium, until cells reached 60–70% confluence. In S9 and IB3-1 cells, the CFTR activity was stimulated by adding to the cells 200 µM dibutyryl cAMP, 200 µM IBMX and 20 µM isoproterenol, in serum-free medium for 24 h.

**Figure 4:**
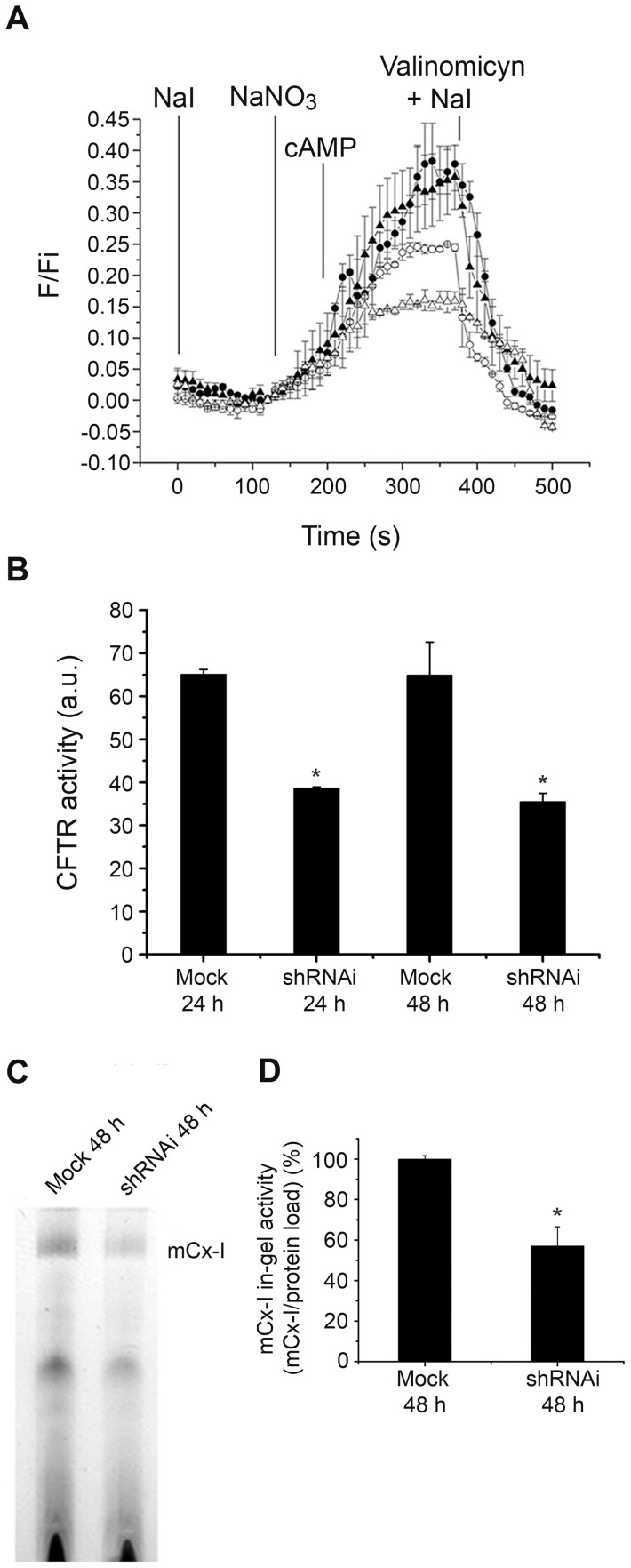
Effects of CFTR-shRNAi in T84 cells on the mitochondrial complex I in-gel activity (IGA). The figure shows the CFTR channel transport activity of T84 cells transiently transfected with a shRNAi plasmid against *CFTR* and its effects on the IGA of mCx-I. A: T84 cells were transfected with empty plasmids (as control, mock-transfected cells) and shRNAi plasmids, to transiently knock down CFTR. Transfected cells were loaded overnight with 5 mM SPQ (Cl^−^ fluorescent probe) to measure CFTR chloride transport activity. The CFTR activity was measured 24 h (shRNAi: -○-, mock: -•-) and 48 h (shRNAi: -Δ-, mock: -▴-) post electroporation. NaI, indicates perfusion with buffer NaI to quench the SPQ fluorescence at the beginning of the experiment. NaNO_3_, indicates the addition of the NaNO_3_ buffer to measure the basal activity of the CFTR. cAMP, indicates stimulation of the CFTR activity by adding 200 µM cAMP, 10 µM isoproterenol, 200 µM IBMX in NaNO_3_ buffer. NaI plus Valinomicyn, indicates the addition of quenching buffer. F, indicates fluorescence values; Fi, are initial fluorescence values just before adding the NaNO_3_ buffer. B: To analyze the CFTR activity changes observed in panel A, the halide efflux was expressed as the area under the curve (integration), expressed as arbitrary units (a.u.). Mock: T84 cells transfected with the empty plasmid as control; shRNAi: T84 cells transfected with the shRNAi plasmid, (24 h and 48 h post transfection). Data are expressed as mean ± SE of two independent experiments (n = 2). * indicates p<0.05 as compared with mock-transfected cells. C: IGA of the mCx-I from mitochondrial extracts corresponding to T84 cells transfected with CFTR-specific shRNAi or empty pSilencer plasmids (Mock). Measurement was performed 48 h post transfection. D: Densitometric quantification and statistical analysis of the results shown in panel C indicated as the ratio (mCx-I activity)/(protein load). Measurements were performed in duplicate and data are expressed as mean ± SE of four independent experiments (n = 4). * indicates p<0.05, as compared with mock-transfected cells (ANOVA and Turkey's test).

### Inhibition of the CFTR chloride transport activity

T84 cells were cultured in 150 cm^2^ tissue culture dishes until they reached 60–70% confluence. At that point, the culture medium was removed and serum-free medium was added to the cells. After 24 h incubation at 37°C, 100 μM glibenclamide, 5 μM CFTR(inh)-172 or 5 µM GlyH101 was added to the serum-free medium and the cells were incubated at 37°C for additional 24 or 48 h (the culture medium containing the inhibitors was renewed after 24 h of incubation; also the medium from controls cells was replaced). Stock solutions for each inhibitor were prepared at 1000 X in DMSO and control cultures were treated with equal amounts of DMSO (final concentration 0.1–0.3%).

**Figure 5:**
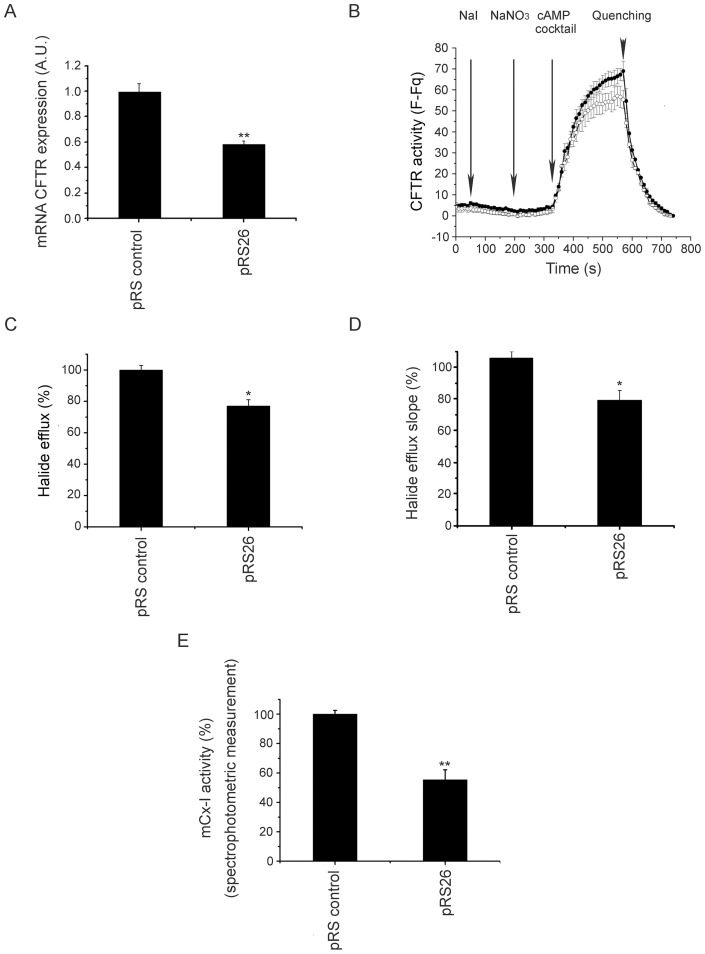
Stable CFTR knock down and mCx-I activity. A: CFTR mRNA expression levels in Caco-2/control cells (transfected with pRS control) and Caco-2/pRS26 cells (transfected with the shRNAi pRS26) determined by quantitative real-time RT-PCR. The results were expressed in arbitrary units (a.u.). Measurements were performed in five independent experiments (n = 5) each done in duplicate. B: CFTR channel halide transport activity of pRS control (-•-) and pRS26 cells (-○-). Arrows indicate the points of buffers addition. F, indicates fluorescence values; Fq, are the fluorescence values after SPQ quenching by adding NaI plus valinomicyn buffer (at 750 s). The graph is representative of three independent experiments (n = 3), each done in duplicate. Changes in the halide efflux between pRS control and pRS26 cells, where represented as the areas under the curve (total halide efflux, panel C) and also as the halide efflux slopes (slope of the first 10 points after cAMP stimulation, adjusted by linear regression) (halide efflux rate, panel D). C and D data were plotted as percentage (%) relative to controls. E: Spectrophotometric measurement of the mCx-I activity in CFTR knock down cells compared to control cells, expressed as percentage (%) relative to control values. The cells were incubated 24 h in serum free medium before the experiments. All data were expressed as mean ± SE. ** indicates p<0.001 and *p<0.05, as compared with control cells. Statistical analyses were performed by ANOVA and Turkey's test.

### Mitochondria isolation

Mitochondria were isolated by using differential centrifugation, according to a slightly modified version of the method described by Majander et al. [33]. Briefly, cells were washed with PBS, scrapped and sedimented by centrifugation (500× g, 10 min). The pellet was then resuspended in isolation buffer (250 mg of cells per ml of buffer containing 0.25 M sucrose, 25 mM MOPS and adjusted to pH 7.4 with KOH) and the cells permeabilized by adding 0.12% w/v digitonin (stock solution 10 mg/ml in water) for 40 s on ice. Then, the samples were diluted in three volumes of isolation buffer and centrifuged at 10,000× g for 30 min (4°C). The pellet was resuspended again in 800 µl of isolation buffer and centrifuged at 800× g for 5 min to discard nuclei and whole cells. Then, the supernatant was centrifuged at 10,000× g for 15 min (4°C) to recover mitochondria. This final pellet was resuspended in 30–50 µl of Blue Native (BN) sample buffer A (1 M aminocaproic acid, 150 mM bis-Tris-HCl, 10 µM pepstatin, 10 µM leupeptin, 100 µM PMSF, 1 mM EDTA, pH 7.0). Finally, the method of Lowry was used to measure protein concentration in aliquots previously incubated with 0.4 N NaOH for 30 min at RT, to dissolve mitochondrial membranes [44].

**Figure 6:**
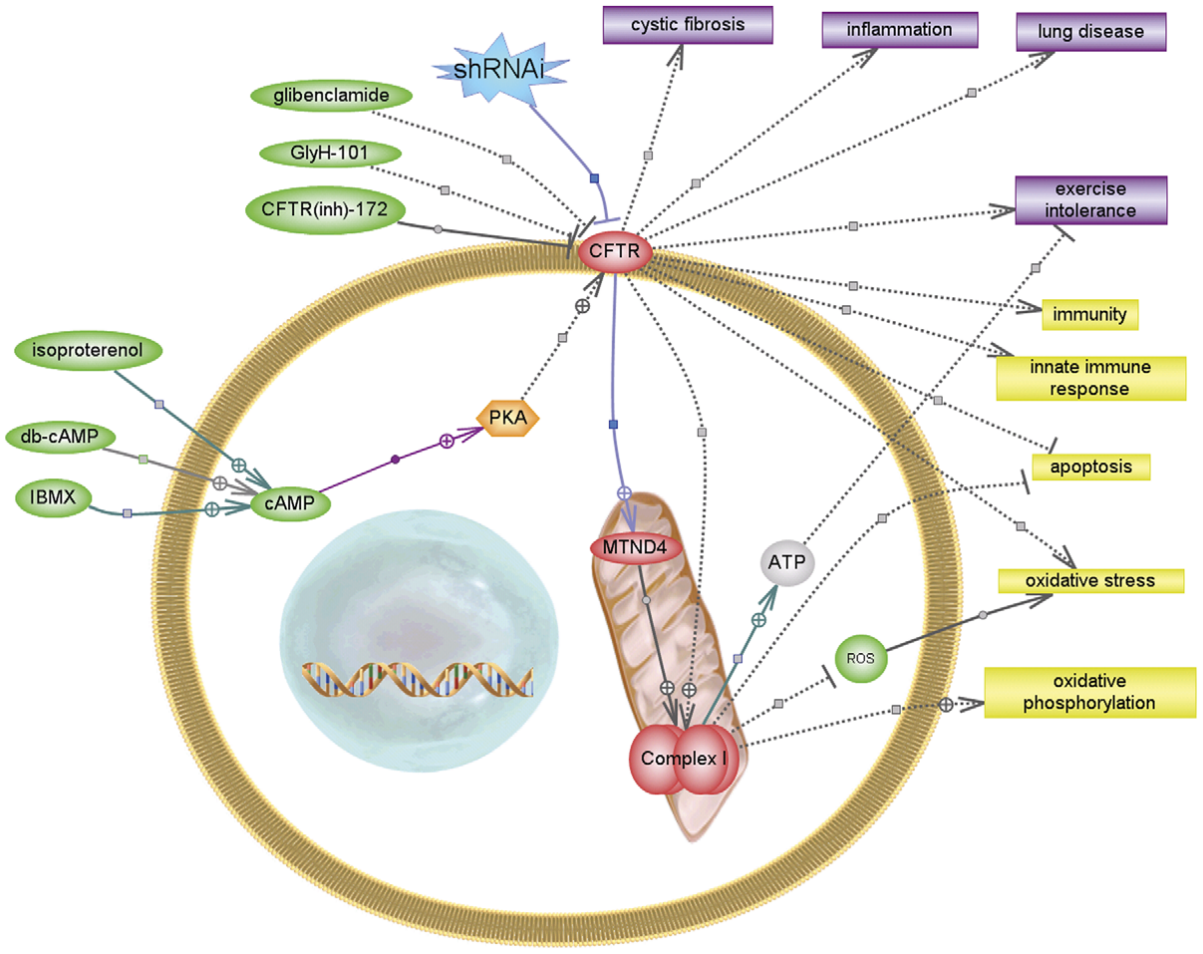
CFTR modulation and reduced mCx-I activity. The graphic illustrates the results obtained and possible effects of a reduced activity of mCx-I, according to know relationships extracted from published work by using the Pathway Studio Software (Ariadne Genomics). Small molecules are indicated in green, proteins in red-orange, cellular processes in yellow and diseases in violet. Some relationships found by the program through its curated database were deleted or fused to simplify the illustration and few were added manually using data extracted from PubMed by using the program subroutines (the last relationships shown as solid lines).

### Blue Native-PAGE

Mitochondrial membranes containing the respiratory chain complexes were solubilized according to Schägger et al. [45], with few modifications. Aliquots containing 150 µg of mitochondrial protein were resuspended in BN sample buffer A to a final protein concentration of 3 µg/µl. Protein complexes were solubilized at 4°C using lauryl maltoside (final concentration 0.6% w/v), and the mixture was then centrifuged at 25,000× g for 30 min at 4°C. The supernatants were collected and protein concentration was measured by Lowry. Before loading the gel, BN sample buffer B (1 M aminocaproic acid, 20% glycerol, 150 mM bis-Tris-HCl, 5% w/v Coomassie brilliant blue G-250, pH 7.0) was added to samples containing equal quantities of protein (60–100 µg in different experiments; usually 80 µg) [46]. The ratio (BN sample buffer B)/sample was 1∶14. Samples were electrophoresed in a 5–13% BN-PAGE (Blue Native PAGE) gradient gel with a 4% stacking gel. The gels and electrophoresis buffers were prepared as described by Schägger [45]. The gel, the buffers and the electrophoretic chamber were cooled to 0–4°C (ice/water) before loading the samples and maintained at the same temperature range during the run. Molecular weight standards were prepared as indicated by the manufacturer (high molecular weight calibration kit, Amersham Biosciences, Buckinghamshire, England). Electrophoresis was carried out at 80 V, 4°C until protein samples migrated into the stacking gel. Voltage was then set to 200 V and the current limited to 15 mA. Once the trace dye had migrated half-way into the separation gel, the cathode (−) buffer (50 mM Tricine, 15 mM bis-Tris-HCl, 0.02% Coomassie brilliant blue G-250, pH 7.0) was replaced with the same buffer without the G-250 dye. This procedure helps to reduce the dye background, which might prevent the detection of the mCx-I activity at the end of the run.

### Measurement of the in-gel mCx-I activity (IGA)

Following electrophoresis, to measure the in-gel activity (IGA) of mCx-I, the gel was incubated (protected from light) for 40 min in a buffer containing 0.1 M Tris-HCl, 0.14 mM NADH, and 1.22 mM NBT, pH 7.4 [47]. A fixing solution containing 45% methanol:10% acetic acid was then used to stop the reaction and the gels were distained overnight (ON) in the same solution, to remove the Coomassie background. The gels were then scanned (HP ScanJet G3110 scanner) and the signal intensities quantified by densitometry using the NIH Image software (Windows version, Scion Corp., Frederick, MD), or its Java version (ImageJ, rsbweb.nih.gov). As a first approach to control sample loads, the gels were stained (0.25% Coomassie brilliant blue R-250, 40% methanol, 7% acetic acid) for 1 h under constant shaking and then distained ON (10% acetic acid and 10% methanol), scanned, and quantified. The IGA of mCx-I was quantified by densitometry (HP scanner and ImageJ) to obtain the relative areas of the peaks. The results were expressed as the ratio of IGA/(total protein content) measured by Lowry (not shown) or by quantifying the gel bands after Coomassie staining (shown in results). Both procedures give similar results. In some assays we used the mitochondrial Complex IIII (mCx-III)(its UQCRC1 subunit or ubiquinol-cytochrome c reductase core protein I), as an internal standard, quantified by using Western blotting and densitometry, performed after the BN-PAGE run, as indicated below.

### Western blotting

For a more specific loading control, in some cases the BN-PAGE proteins corresponding to the lower half of the gel (the upper half was simultaneously used to measure the IGA as above indicated) were transferred to PVDF (polyvinylidene difluoride) membranes using transfer buffer without methanol (39 mM glycine, 48 mM Tris-base, 0.037% SDS, pH 8.3), 4 h at 100 V, constant voltage. Membranes were blocked with 5% defatted milk in PBS and incubated with a monoclonal antibody raised against the ubiquinol-cytochrome c reductase core protein I (UQCRC1), a core subunit of the mitochondrial complex III (mCx-III, EC 1.10.2.2) (Santa Cruz Biotechnology, CA; sc-65238, dilution 1∶700 in PBS plus Tween 20, 0.05% v/v), used as internal control for sample loading and as a reference for mCx-I changes. After 1 h incubation with the primary antibody, the membranes were washed three times with PBS plus Tween 20, 0.05% v/v for 5 min. Then, the membranes were incubated for one hour with a secondary goat IgG anti-mouse antibody (Santa Cruz Biotechnology, sc2008, dilution 1∶2500) coupled to alkaline phosphatase, washed three times with PBS plus Tween 20 0.05% v/v for 5 min and developed with the substrates NBT-BCIP following the manufacturer's instructions (Promega).

### Spectrophotometric measurement of mitochondrial NADH-cytochrome c reductase (mCxI-mCxIII) activity

The mitochondria were isolated as previously described [48], [49] and were subjected to three freeze-thaw cycles to make them permeable to substrates. To measure the activity of Complex I, 100 µg mitochondria were resuspended in buffer solution (100 mM H_2_KPO_4_/HK_2_PO_4_, 0.5 mM KCN, 200 µM NADH, 25 µM cytocrome c_,_ pH 7.4) and the reduction of cytocrome c was recorded by monitoring the increase in absorbance at 550 nm (E = 19 mM-1 cm-1), 30°C. The mCx-I activity was calculated as nmol cytochrome c reduced/min/mg protein, and expressed in percentage considering the activity in control cells as 100%. Inhibition of mCxI by rotenone was measured after 5 min of pre-incubation with the inhibitor and subtracted to the values in the absence of rotenone incubation.

### shRNAi preparation for transient transfection

To specifically and transiently knock-down CFTR expression, we prepared a shRNAi (short hairpin RNA interference) by inserting into a pSilencer 1.0 U6 vector (Ambion, Austin, TX) the same sequence previously used as an antisense oligonucleotide, which is complementary to nucleotides 1–18 of the CFTR mRNA [17], [50]. The antisense sequence and its complementary strand plus a small connecting loop were synthesized by using an Oligo1000M DNA synthesizer (Beckman-Coulter, Fullerton, CA). To construct the insert, two complementary oligonucleotides containing *Eco*RI and *Hind*III restriction sites were added at the 5′and 3′ends of the DNA oligonucleotide, and also a connecting loop of sequence TTCAAGAGA, following the recommendations from Ambion (Oligo 1 *Hind*III: 5′-AGCTTCATGCAGAGGTCGCCTCTGTTCAAGAGACAGAGGCGACC TCTGCATGTTTTTT-3′; Oligo 2 *Eco*RI: 5′-AATT AAAAAACATGCAGAGGTCGCCTCTG TCTCTTGAACAGAGGCGACCTCTGCATG-3′; the target sequence is underlined). These oligonucleotides were then annealed and cloned into the *Eco*RI and *Hind*III restriction sites of the pSilencer 1.0 U6 vector. Finally, the plasmid was sequenced to check for the proper insertion by using an institutional core sequencing facility (at the Institute L.F. Leloir, Buenos Aires).

### shRNAi transient transfections

T84 cells cultured in DMEM/F12 medium containing 10% FBS and maintained below 70% confluence were collected by using 0.5% Trypsin/PBS and electroporated by using a BTX ECM 830 square-wave electroporator (Genetronics Inc., San Diego, CA) and electroporation cuvettes (Genetronics Inc. or Bio-Rad Laboratories) for mammalian cells (electrode gap 0.4 cm). The electroporation settings were “low voltage” mode at 140 volts and 1 pulse of 70 ms. In order to set up the transfection conditions, different amounts of shRNAi plasmid were used (20, 40 and 80 µg). The optimal amount of plasmid rendering reproducible results towards mCx-I inhibition was 40 µg/4×10^6^ cells, in a final volume of 400 µl of serum-free DMEM/F12. This amount was therefore used in subsequent experiments. In addition, to determine the optimal culture time to inhibit mCx-I expression, cells were cultured in serum-free DMEM/F12 for 24, 48 and 72 h after shRNAi transfection. Under these conditions, the optimal culture time was 48 h and this time was then used for the subsequent assays.

### shRNAi stable transfections

To specifically knock-down CFTR expression, we used four short hairpin RNA interference (shRNAi) against four different regions of CFTR. The knock down sequences were inserted for the manufacturer (OriGene Technologies, Inc., Rockville, USA) as shown below: Human U6 promoter – GATCG –29 nt sense – TCAAGAG –29 nt reverse complement – TTTTTT -3′. The four sense sequences were pRS25: AAGAAATA TGGAAAGTTGCAGAT GAGGTT; pRS26: AAATATCATCTTTGGT GTTTCCTATGATG; pRS27: ACAACTGGAATCTGAAGGCAGGAGTCCAA; and pRS28: CTTACTTTGAAACT CTGTTCCACAAAGCT. The sequence corresponding to pRS-shGFP was used as a control, corresponding to a non-effective shRNA plasmid against GFP, provided by OriGene. Caco-2 cells were cultured in DMEM/F12 medium containing 10% FBS and maintained below 70% confluence. Then, cells were collected by trypsin (0.25% trypsin, 0.02% EDTA in PBS) treatment and electroporated using a BTX ECM 830 square-wave electroporator (Genetronix Inc., San Diego, CA). The electroporation was performed by using a cuvette plus (Genetronix Inc., San Diego, CA) for mammalian cells. Settings for electroporation were 140 volts and 1 pulse of 70 msec, using 40–100 μg of shRNAi plasmid and 4×10^6^ cells, in a final volume of 400 µl. Transfected cells were selected in DMEM/F12 plus 10% FBS, containing 4 µg/ml of Puromicin, for 20 days. To increase the plasmid concentration by cell, the Puromicin was gradually incremented up to 20 µg/ml and then returned to 5 µg/ml. Selected cells were cloned by limit dilution in 96 well plates. Then, clones with high CFTR knock-down were selected by dot blot analysis with a monoclonal CFTR antibody prepared in our laboratory, which has similar specificity and sensitivity compared to the monoclonal Ab prepared by John Riordan (unpublished results). To perform the mCx-I assay, cells were plated at a density of 3×10^3^ cells in 150 cm^2^ tissue culture dishes and grown in DMEM/F12 containing 10% FBS cand Puromicin 1 µg/ml, at a confluence of 70–80%. Then, the cells were cultured 24 h in serum-free medium.

### CFTR transport activity in shRNAi-transfected cells

The fluorescent probe SPQ (6-methoxy-N-[3-sulfopropyl]quinolinium) was used to measure the CFTR chloride transport activity, as SPQ fluorescence is quenched by chloride and other halides, such as iodide and bromide. A slightly modified version of the method described by Verkman's laboratory [51], [52] was used, as described recently [53]. Briefly, a Hitachi's slice holder was adapted to form a perfusion chamber, allowing to measure CFTR activity by using fluorescence spectrophotometry. T84 cells freshly transfected with shRNAi and mock (control) plasmids were grown in p60 plates (0.3×10^4^ cells/cm^2^) containing at least 2 rectangular coverslips (22×8 mm, from Hitachi). The coverslips were pre-treated with a coating solution (10 µg/ml fibronectin, 4.4 µg/ml collagen, 1.5 µg/ml BSA in DMEM/F12) for one h at 37°C and washed with serum free medium. After transfection, the cells were cultured for 24 or 48 h over the coverslips in DMEM-F12 serum-free medium. The cells were then incubated ON in 5 mM SPQ (dissolved in serum-free DMEM/F12) , washed three times with NaI buffer (135 mM NaI, 10 mM Glucose, 1 mM CaSO_4_, 1 mM MgSO_4_, 10 mM Hepes, 2.4 mM K_2_HPO_4_, and 0.6 mM KH_2_PO_4_, pH 7.4 ) and maintained at 37°C for 30 min. Each coverslip was then placed in a separated culture dish and maintained under light-protecting conditions. For measurements, the coverslips containing confluent monolayer cells were inserted in a holder specially designed by Hitachi for the F2000 spectrophotometer, and immersed into a quartz cuvette containing NaI buffer, inside the fluorescence spectrophotometer. The coverslip holder was previously modified with a drill to allow the insertion of two tubes of different diameter and length [53]. These tubes, coupled to a peristaltic pump, were used to perfuse the quartz chamber. All measurements were carried out at 37°C, under perfusion and stirring. The selected wavelengths for SPQ were Ex  = 344 nm and Em  = 443 nm. To measure the baseline fluorescence (Fb), cells were perfused with NaI buffer for 100 s. Then, the cells were sequentially perfused with the NaNO_3_ buffer (135 mM NaNO_3_, 10 mM Glucose, 1 mM CaSO_4_, 1 mM MgSO_4_, 10 mM Hepes, 2.4 mM K_2_HPO_4_, 0.6 mM KH_2_PO_4_, pH 7.4), a cocktail containing CFTR activators (buffer NaNO_3_ containing 200 µM dibutyryl cAMP, 200 µM IBMX and 20 µM isoproterenol) and the quenching buffer (5 µM valinomycin in NaI buffer). Perfusion times were 200 s, 200 s and 100 s, respectively. The stock solutions of valinomycin, IBMX, and dibutyryl cAMP were prepared at 1000 X in culture-grade DMSO (Sigma-Aldrich). Isoproterenol was dissolved in water at 1000 X concentration. The collected data were plotted as F/Fi –1 vs time (F: fluorescence; Fi: initial fluorescence when the NaNO_3_ buffer was added). Later, F-Fq vs time (F: fluorescence; Fq: fluorescence value obtained after quenching the SPQ fluorescence by adding NaI plus valinomycin, at the end of SPQ fluorescence quenching) was preferred, since the basal activity Fi was different between the different analyzed cells whereas the background fluorescence Fq was very reproducible. The software Origin (Originlab Corp., Northampton, MA) was used to integrate the area under the curve and to obtain the slopes at initial times of stimulation.

### Real-time RT-PCR (RT-PCR)

To determine the levels of *CFTR* mRNA knock-down obtained by shRNAi transfections, real-time PCRs (RT-PCR) were performed and the ΔΔCt method used for comparative quantification. Total RNA samples (4 µg) derived from Caco-2 clones transfected with four different shRNAi specific for CFTR and one shRNA control (shRNA for GFP) were used for reverse transcription by using M-MLV Reverse Transcriptase (Promega) and Oligo-dT, according to the manufacturer's instructions (100 U of RT/μg of RNA). Quantitative RT-PCR was carried out using the expression of *TBP* as an internal control. The primers were designed with Primer-BLAST software. Primer sequences for *CFTR* were: Rv-*CFTR* 5′-TGGTCTGGTCCAGCTGAAAAA-3′; Fw-*CFTR* 5′-GTAGGTCTTTGGCATTAGGAGCTT-3′; and the primers for *TBP* were Rv-*TBP* 5′-C ACATCACAGCTCCCCACCA-3′; and Fw-*TBP* 5′- TGCACAGGAGCCAAGAGTG AA-3′. The size of each amplification product and the presence of dimers were verified by electrophoresis on a 3% agarose gel, stained with ethidium bromide and visualized by using UV. Then, preliminary experiments with different cDNA dilutions were performed to test the dynamic range and efficiency of amplification for each amplicon (*TBP* and *CFTR*). The cDNA samples (10 µl of a 1∶50 cDNA dilution) were added to 15 μl of PCR reaction mixture containing a final concentration of 2.5 mM MgCl_2_, 0.4 mM deoxynucleotides triphosphates, 1 U of Go*Taq* DNA polymerase (Promega), 0.1 X EvaGreen (Biotium, Hayward, CA), 50 nM ROX (SIGMA) as passive reference dye and 0.2 nM of each primer. qRT-PCR reactions were carried out in an Applied Biosystems 7500 Real-Time PCR equipment. PCR conditions were: denaturation at 94°C (5 min), and 40 cycles of 94°C (30 s), 60°C (30 s), and 72°C (30 s). qRT-PCR reactions were carried out in technical (intraassay) and biological triplicates. The final quantification values were obtained as the mean of the Relative Quantification (RQ) for each biological triplicate (n = 3).

### Statistics

Unless otherwise indicated, all assays were performed in triplicates, the experiments were repeated at least three times and the results expressed as mean ± SE (n =  replicates). One-way ANOVA and the Turkey's test were applied to determine significant differences among samples (α = 0.05).

## Results

### The mCx-I in-gel activity (IGA) of CFDE and CFDE/6RepCFTR cells

To test the hypothesis of a reduced mCx-I activity in CF cells, mitochondrial extracts from CFDE and CFDE/6RepCFTR cells cultured 24 h in serum-free medium, were run under Blue-Native PAGE (BN-PAGE) to determine the IGA of mCx-I [45], [46], [54]. CFDE cells are tracheobronchial cells derived from a CF patient and CFDE/6RepCFTR cells are CFDE cells ectopically expressing CFTR [38]. As shown in Figure 1, the IGA of mCx-I was significantly (p<0.001) reduced in CFDE cells (47.0±2.1%; mean ± SE, n = 3) as compared with the wt-CFTR-complemented CFDE/6RepCFTR cells (104.9±4.9%, n = 3). As a control for CFTR specific effects, CFDE/6RepCFTR cells were also treated with 100 µM glibenclamide (a CFTR chloride channel inhibitor) for 24 h, in serum-free medium. As shown in Figure 1A (IGA) and 1B (quantification), a significant (p<0.01) reduction of the IGA of mCx-I was observed (71.3±3.9%, n = 3) as compared with CFDE/6RepCFTR cells not treated with the inhibitor (104.9±4.9%, n = 3). These results suggest a causal relationship between the chloride transport activity of CFTR and the mCx-I, and are in agreement with our previous observation showing that the expression of *MTND4* is reduced in CFDE cells [15].

### The mCx-I in-gel activity (IGA) of IB3-1 and S9 cells

To make sure that the differences in the mCx-I activity observed between CFDE and CFDE/6RepCFTR cells did not resulted from an artifact created by a different selection pressure (due to the antibiotic used to select for CFDE/6RepCFTR cells, the different passage number between the two cell lines or a randomly favored clonal selection), we also measured the IGA of mCx-I using the cell lines IB3-1 and S9, unrelated to CFDE cells. The IB3-1 cells were derived from a CF patient exhibiting the most frequent CF mutation (ΔF508) in one allele and a non-sense mutation (W1282X) in the other allele [39]. S9 cells are IB3-1 cells transduced with an adeno-associated viral vector to stably express wt-CFTR [40]. Thus, antibiotics were not required to maintain the expression of wt-CFTR in S9 cells. As shown in Figure 2A (IGA) and 2B (quantification), under basal conditions, no significant differences were observed on the IGA of mCx-I between IB3-1 and S9 cells. However, when IB3-1 and S9 cells were treated for 24 h with a CFTR-stimulating cocktail (200 μM cAMP, 200 μM IBMX and 20 μM isoproterenol), a significant (p<0.05) and reproducible difference on the IGA was observed between IB3-1 cells (54.4±6.9%, mean ± SE, n = 5) and S9 cells (100.5±3.7%, n = 5). Thus, under CFTR-stimulation, the IGA of mCx-I in IB3-1 CF cells was almost 50% lower than in wt-CFTR complemented IB3-1 cells (S9 cell line).

The IGA of mCx-I was also measured by using the relative UQCRC1 amounts as an internal standard (indicative of mCx-III) (IGA of mCx-I/UQCRC1 amounts; Figure 2C and 2D), instead of total protein load. In this case the IGA of mCx-I was also significantly reduced in IB3-1 cells (72.2±10.7%; mean ± SE, n = 4) compared to S9 cells (100.0±10.2%, n = 4), although the difference obtained was smaller. Taken together, these results indicate that using either CFTR stimulation or inhibition to modify the CFTR activity, a significant modulation of the IGA of the mCx-I can be observed, and add further support to the results initially obtained with CFDE cells (shown in Figure 1). The relative specific activity (expressed as the ratio between mCx-I IGA and the Coomassie blue staining shown in Figure 2E) is shown in f panel 2F. In this case, no significant differences were observed between IB3-1 (CF) and S9 (CF corrected) cells, suggesting that the differences in mCx-I in gel activity reflect a reduction in the amount of mCx-I rather than a difference in the specific activity of Complex I.

### In gel activity of mCx-I measured in cells expressing wt-CFTR

To add further support to the results, we next used non-CF cells, in which the activity or expression of CFTR was modulated by using CFTR inhibitors or shRNAs. For this purpose, T84 and Caco-2 colon carcinoma cells were used; these cells express high levels of CFTR [41], [42], [55].

As shown in Figure 3A (IGA) and 3B (quantification of 3A), when T84 cells were cultured for 24 h in the presence of glibenclamide (100 µM), the IGA of mCx-I was significantly (p<0.05) reduced to 56.8±8.2% (mean ± SE, n = 3) as compared with control cells (100.1±0.12%, n = 3). When T84 cells were cultured for 24 h in 5 μM CFTR(inh)-172 (a more specific and potent CFTR inhibitor than Glibenclamide [56]) no changes were observed in IGA of mCx-I (Figure 3A and 3B). However, as shown in Figure 3C (IGA) and 3D (quantification of 3C), after 48 h of incubation with CFTR(inh)-172, the IGA of mCx-I was significantly (p<0.05) reduced (64.4±4.1%, n = 3) compared to control T84 cells (100.0±0.06%, n = 3). The IGA corresponding to the glibenclamide treatment for 48 hours shown a significant reduction (Figure 3C and 3D); however, under these incubation conditions (100 µM glibenclamide for 48 hours) this inhibitor was probably toxic for these cells since the cells started to detach and the medium became acid.

To assure that these results were not limited to T84 cells, we then treated Caco-2 cells with 5 μM CFTR(inh)-172. In addition, to diminish the possibility of a nonspecific effect of CFTR(inh)-172, an additional CFTR inhibitor was now used, GlyH101, at 5 µM for 48 h. The GlyH101 inhibitor has a better solubility in water compared with CFTR(inh)-172 [57]. As shown in Figure 3E (IGA) and 3F (quantification of 3E), the IGA of mCx-I (IGA of mCx-I/UQCRC1 amounts) was significantly (p<0.05) reduced in Caco-2 cells treated with CFTR(inh)-172 (41.7±8.6%; mean ± SE, n = 3) or treated with GlyH101 (61.9±11.0%, n = 3) compared to control cells (100.0±20.9%, n = 3).

### Transient shRNAi-mediated knock down of CFTR expression in T84 cells

To reduce the possibility that the results obtained after treating cells with glibenclamide, CFTR(inh)-172 or GlyH101 resulted from nonspecific effects of these drugs [58], shRNAi was used to knock-down the CFTR expression in T84 cells (CFTR-shRNAi). SPQ fluorescence was used to verify that the CFTR chloride channel activity was in fact reduced in the shRNAi-transfected cells (Figure 4A). The Figure 4A shows that the CFTR activity was reduced in cells transfected with shRNAi for CFTR (shRNAi-CFTR) as compared to cells transfected whit empty plasmid, 24 h and 48 h post electroporation. In Figure 4B, the changes in the CFTR activity observed in Figure 4A were represented as the areas under the curves (numerical integration). As shown in Figure 4B, 24 h after shRNAi transfection, a significant (p<0.05) reduction in the CFTR chloride transport activity was observed in T84 cells (38.6±0.2 a.u., mean ± SE, n = 2)(a.u. or A.U.: arbitrary units), as compared with mock-transfected cells (transfected with empty plasmid) (65±1.1 a.u., n = 2). This inhibition was slightly more pronounced 48 h after transfection (35.5±1.9 a.u., n = 2); therefore, all subsequent experiments were performed 48 h after transfection. The Figure 4C shows the IGA of mCx-I after transfecting T84 cells with 40 µg of CFTR-shRNAi plasmid, 48 h post-electroporation. The IGA of mCx-I in shRNAi-transfected cells was significantly (p<0.05) lower (57.0±9.5%, mean ± SE, n = 4) than in mock-transfected cells (100.0±1.6%, n = 4). The shRNAi effects are in agreement with the results obtained after glibenclamide, CFTR(inh)-172 or GlyH101 treatments, and further support the idea that the CFTR activity modulates the mCx-I activity.

### Stable shRNAi-mediated knock down of CFTR expression in Caco-2 cells

The results obtained by using transient transfection of shRNAi for CFTR were in agreement with the mCx-I decrease observed previously. However, the transient transfections were difficult to reproduce due to low transfection efficiencies. To overcome this problem, Caco-2 cells were transfected with 4 commercial shRNAi plasmids directed against CFTR and selected by using Puromicine. Four commercial plasmids against different regions of CFTR mRNA (named pRS25, pRS26, pRS27 and pRS28) and a control plasmid (named pRS control) were used. The cell line with better CFTR knock down (Caco-2/pRS26 cells) was selected to perform the mCx-I analysis. The CFTR mRNA expression was analyzed by qRT-PCR, and, as shown in Figure 5A, Caco-2/pRS26 cells shows a highly significant (p<0.001) decrease in the CFTR levels (0.58±0.03 a.u.; mean ± SE; n = 10) compared to control cells (0.99±0.06a.u., n = 10). To corroborate the CFTR knock down in these cells, the activity of CFTR was measured by using the chloride sensitive probe SPQ (Figure 5B). As shown in Figure 5C, the halide efflux (area under the curves) was significant (p<0.05) reduced in Caco-2/pRS26 cells (77±3.99%; mean ± SE; n = 6) compared to control cells (100±2.9%; n = 6). Similar results were obtained for the halide efflux slopes using the first 10 points after the CFTR stimulation and adjusted by linear regression (Figure 5D), which also reflect a lower CFTR concentration in Caco-2/pRS26 cells than in control cells. Finally, the Figure 5E, shows the spectrophotometric analysis of the mCx-I/mCx-III activity for the CFTR knock down cell lines. A highly significant (p<0.001) reduction was observed (55.3±6.7%; mean ± SE; n = 5) as compared to controls (100±2.5%; n = 5). These spectrophotometric measurements are in agreement with the IGA results.

While this work was in progress, Kelly-Aubert et al, studying the effects of a glutathione analog (GSH monoethyl ester), also found a reduced mCx-I activity in CF cells and KO mice [59], in agreement with our previous findings regarding a reduced *CISD1* [14] and *MTND4* [15] expression in CF cells, which prompted us to think again over the mitochondrial hypothesis of Shapiro and colleagues [10], [11], [12], [34], [35], [36], [37]. However, contrary to Kelly-Aubert, which found similar amounts of total mCx-I and therefore increased specific activity of mCx-I in normal cells (or reduced in CF cells), we found a similar specific activity in both cases, as shown in Figure 2 E and 2F and a reduced mCx-I amount.

## Discussion

The results shown here suggest that the mitochondrial Complex I activity is positively modulated by the chloride transport activity of CFTR (or negatively regulated under inhibition of CFTR activity or expression). This regulation was observed using different cellular models, including cells from CF or non-CF origin. A significant reduction in the in-gel activity (IGA) of mCx-I (near 50%) was observed in CFDE cells (CF-derived cells) as compared with CFDE/6RepCFTR (wt-CFTR complemented cells). This effect was reverted in CFTR complemented cells treated with the CFTR inhibitor glibenclamide. The inhibitory effect of glibenclamide over the mCx-I activity suggests that the differences observed between CFDE and complemented CFDE cells are not just a simple correlation resulting from some epiphenomena caused by unspecific clonal selection, antibiotic treatment, differences in growth speed, or some other unknown effects. Thus, the reduction in the mCx-I activity under CFTR inhibition suggests that a causal relationship exists between the chloride transport activity of CFTR and the IGA of mCx-I.

A similar effect (reduced IGA in CF cells) was observed using IB3-1 and S9 cells (the last are wt-CFTR complemented IB3-1 cells), even though CFTR-stimulation instead of its pharmacological inhibition was used in this case, effects corresponding to different molecular mechanisms (PKA phosphorylation of the CFTR domain R vs. blocking of the channel transport activity). The hypothesis that CFTR activity or expression can modulate the mCx-I activity was further supported using cells that naturally express wt-CFTR (T84 and Caco-2 cells). In these cells, the CFTR activity was inhibited by incubation with glibenclamide, CFTR(inh)-172, GlyH101 or through transfection with a shRNAi, obtaining similar results in each case. Thus, the observed effects on the mCx-I activity cannot be attributed to nonspecific effects of the pharmacological inhibitors used, since similar effects were obtained by using shRNAi, which is using yet another mechanism (RNA degradation vs. chloride transport inhibition or activation). Noteworthy, the inhibition of the Cl^−^ transport activity was less pronounced that the inhibition of the mRNA levels in shRNAi treated cells (Figure 5). Also, a relatively small inhibition in the CFTR chloride transport activity on shRNAi cells (∼23%) produced a more pronounced effect on the mCx-I activity (∼45%) (Figure 5). Similar responses has been previously observed by MacVinish et al. [60], having over 95% inhibition on CFTR mRNA content in airway epithelial Calu-3 cells treated with stable RNAi but only show a 25% reduction in the CFTR Cl^−^ transport activity compared to controls. The authors suggest that an intracellular pool of CFTR might exists which change with RNAi treatment, but only a small fraction of CFTR on the membrane is actually affected [60].

In conclusion, the results suggest the existence of a causal relationship between the CFTR chloride transport activity and the mCx-I activity. In addition, the effect on the mCx-I activity appear to be dependent on the CFTR chloride transport activity and not only due to the presence/absence of the CFTR in the cell membrane, as reported for the expression of the chemokine RANTES [61], which responds to the presence of the CFTR in the cell membrane, being insensitive to pharmacological inhibitors of CFTR.

In a previous work, we have shown that the expression of the *MTND4* gene was reduced by approximately 40% in CF cells or in CFTR-corrected cells treated with CFTR inhibitors (glibenclamide and CFTR(inh)-172), after 24 h of incubation [15]. Here, we show that a similar reduction is observed in the IGA of mCx-I, although the reduction was observed 48 h after treatment with the CFTR inhibitors (CFTR(inh)-172 or GlyH101). Since the reduction of MTND4 expression could be seen earlier than the reduction of the mCx-I IGA (24 h instead of 48 h), the results are also in agreement with the fact that MTND4 is essential for the assembly and activity of mCx-I [29] and suggest a down-stream position of the mCx-I activity compared to the MTND4 expression.

Further studies are required to elucidate the mechanism(s) by which CFTR modulate the activity and expression of CFTR-dependent genes such as c-Src and MUC1 [17], CISD1 [14], MTND4 [15] and now, the mCx-I activity. CFTR-dependent genes and the possible CFTR-signaling effectors are of most interest, since these molecules and their pathways might be potential targets for CF therapy. So far we only know that c-Src is increased in CF and appears to be a bridge between the CFTR channel activity and MUC1 expression [17], and that RANTES expression might be modulated trough interactions involving PDZ binding domains and EBP50 related interactions [61].

Figure 6 summarizes this idea and the possible consequences of a reduced mCx-I activity, according to know relationships extracted by the software Pathway Studio (www.ariadnegenomics.com), using its database or PubMed information. The possible effects of a reduction on the mitochondrial Complex I activity are different and complex [62], including increased ROS production [63], increased apoptosis [64], reduced ATP synthesis [65], and even alterations on innate immunity [66]. Interestingly, all these effects have been already reported in CF cells [12], [67], [68], [69], [70], [71]. In the short term, the consequences of a reduced mCx-I activity might not be as evident as the effects observed in the LHON disease (blindness) [31], caused primarily as the result of mutations in mCx-I genes. It is clear that patients with LHON disease do not have susceptibility to lung infections nor CF patients have blindness. Therefore, several concurrent genes should be involved in producing the complex phenotype of CF, with some effects differentially compensated with tissue specificity [72]. A reduced mCx-I activity is probably one additional factor contributing to the complexity of the CF phenotype, although it might be relevant to explain some of the above mentioned mitochondrial defects observed in CF, perhaps including an increased susceptibility to infections [73], [74].
